# Long-term risk of mortality after acute kidney injury in patients with sepsis: a contemporary analysis

**DOI:** 10.1186/1471-2369-11-9

**Published:** 2010-06-02

**Authors:** José António Lopes, Paulo Fernandes, Sofia Jorge, Cristina Resina, Carla Santos, Álvaro Pereira, José Neves, Francisco Antunes, António Gomes da Costa

**Affiliations:** 1 Department of Nephrology and Renal Transplantation, Hospital de Santa Maria, Centro Hospitalar Lisboa Norte, EPE, Av. Prof. Egas Moniz, Lisboa, 1649-035, Portugal; 2 Department of Infectious Diseases, Hospital de Santa Maria, Centro Hospitalar Lisboa Norte, EPE, Av. Prof. Egas Moniz, Lisboa, 1649-035, Portugal

## Abstract

**Background:**

Acute kidney injury (AKI) is associated with increased short-term mortality of septic patients; however, the exact influence of AKI on long-term mortality in such patients has not yet been determined.

**Methods:**

We retrospectively evaluated the impact of AKI, defined by the "Risk, Injury, Failure, Loss of kidney function, End-stage kidney disease" (RIFLE) classification based on creatinine criteria, on 2-year mortality in a cohort of 234 hospital surviving septic patients who had been hospitalized at the Infectious Disease Intensive Care Unit of our Hospital.

**Results:**

Mean-follow-up was 21 ± 6.4 months. During this period, 32 patients (13.7%) died. At 6 months, 1 and 2 years of follow-up, the cumulative probability of death of patients with previous AKI was 8.3, 16.9 and 34.2%, respectively, as compared with 2.2, 6 and 8.9% in patients without previous AKI (log-rank, P < 0.0001). In the univariate analysis, age (hazard ratio 1.4, 95% CI 1.2-1.7, P < 0.0001), as well as pre-existing cardiovascular disease (hazard ratio 3.6, 95% CI 1.4-9.4, P = 0.009), illness severity as evaluated by nonrenal APACHE II (hazard ratio 1.3, 95% CI 1.1-1.6, P = 0.002), and previous AKI (hazard ratio 4.2, 95% CI 2.1-8.5, P < 0.0001) were associated with increased 2-year mortality, while gender, race, pre-existing hypertension, cirrhosis, HIV infection, neoplasm, and baseline glomerular filtration rate did not. In the multivariate analysis, however, only previous AKI (hazard ratio 3.2, 95% CI 1.6-6.5, P = 0.001) and age (hazard ratio 1.4, 95% CI 1.2-1.6, P < 0.0001) emerged as independent predictors of 2-year mortality.

**Conclusions:**

Acute kidney injury had a negative impact on long-term mortality of patients with sepsis.

## Background

Acute kidney injury (AKI) is a common complication among hospitalized patients, particularly in the Intensive Care Unit (ICU) setting, and it portends an ominous outcome. In fact, the incidence of AKI in critically ill patients varies from 35 to 70%, and it is associated with increased in-hospital mortality (10 to 60%) [1-4].

Although AKI is a common complication in the hospital and has an immediate impact on morbidity, mortality, and resource utilization, its detrimental effect appears to persist also after recovery, since AKI has shown to increase long-term mortality [5-7]. Understanding the impact of AKI on long-term outcomes will have a marked impact on treatment and risk stratification during hospitalization and will assist with guiding follow-up care after discharge.

Sepsis is a well-known risk factor for the development of AKI and nearly half of all patients with AKI have sepsis [8,9]. Furthermore, AKI is associated with increased short-term mortality among septic patients [8-14]. Despite the detrimental effect of AKI on short-term outcome, to our knowledge, there is no study evaluating the influence of AKI on long-term mortality of hospital surviving septic patients.

In the present study, we sought to evaluate the impact of AKI on long-term mortality in a cohort of patients with sepsis.

## Methods

This is a retrospective study that evaluated the influence of AKI during previous ICU admission on long-term mortality of hospital surviving septic patients admitted to the Infectious Disease Intensive Care Unit of our hospital between July 2002 and June 2007. This unit is the reference medical infectious disease unit for adult patients with infectious diseases who need intensive care, providing medical assistance to an area (Lisbon to south of Portugal) with almost 3000000 inhabitants.

Since this was a retrospective and observational study that did not evaluate a specific therapeutic or prophylactic intervention the institutional ethical approval was not required according to our institution's guidelines.

### Selection criteria, population and study periods

Between July 2002 and June 2007, 454 patients with sepsis were admitted to the unit; 28 of them were chronic kidney disease patients on dialysis and were not included in this study. None of the patients had received a renal transplant. From the 426 remaining patients (mean age: 53.3 years, 281 male, 379 Caucasian, mean APACHE II score: 21.3) (Table 1), 122 of them died within hospitalization and, therefore, 304 patients were discharged alive from the hospital (Table 2 and Table 3). Mortality was assessed at 2-years of follow-up. For those patients who were followed-up at the hospital with a documented medical visit at or after 2 years after hospital discharge, and for those patients who died at the hospital during a subsequent hospitalization after hospital discharge, long-term outcome was assessed based on hospital records. On the other hand, for those patients who were not followed-up at the hospital, the long-term outcome was assessed by phone call to patients or their family by using contact details provided on hospital database. We could not analyze the long-term outcome of 70 patients who were lost to follow-up. Therefore, in this study, we focused on 234 patients with sepsis, and analyzed their mortality at 2 years of follow-up as a cohort.

**Table 1:** Baseline characteristics of the overall cohort of septic patients.

Variable	Without AKI^1 ^(n = 288)	With AKI (n = 138)	***P***
Mean age (years)	50.7 (18.3)	58.8 (16.4)	< 0.0001

Male (%)	62.8	72.5	0.064

Caucasian (%)	90.6	85.5	0.158

DM^2 ^(%)	14.6	16.7	0.678

Hypertension (%)	20.5	23.9	0.497

CVD^3 ^(%)	6.6	20.3	< 0.0001

Cirrhosis (%)	11.8	10.1	0.731

HIV infection^4 ^(%)	16.3	17.4	0.890

Neoplasm (%)	6.2	11.6	0.087

APACHE II score^5^	18.3 (8)	27.5 (8.6)	< 0.0001

Nonrenal APACHE II score^6^	17.7 (7.8)	23.9 (8.6)	< 0.0001

Baseline SCr (mg/dL)^7^	0.8 (0.2)	1 (0.5)	< 0.0001

Septic shock (%)	22.6	60.1	< 0.0001

MV^8 ^(%)	39.2	59.4	< 0.0001

Length of hospital stay (days)	10.7 (12.5)	12 (13.6)	0.328

In-hospital mortality (%)	15.6	55.8	< 0.0001

^1^AKI-acute kidney injury. ^2^DM-diabetes mellitus. ^3^CVD-cardiovascular disease includes chronic heart failure and coronary artery disease. ^4^HIV infection-human immunodeficiency virus infection. ^5^APACHE II score-acute physiology and chronic health evaluation, version II score. ^6^Nonrenal APACHE II score-acute physiology and chronic health evaluation, version II score not including points for kidney insufficiency. ^7^Baseline SCr-baseline serum creatinine. ^8^MV-need for mechanical ventilation.

**Table 2:** Baseline characteristics of the hospital surviving septic patients.

Variable	Without AKI^1 ^(n = 243)	With AKI (n = 61)	***P***
Mean age (years)	50.2 (18.2)	58.8 (16.3)	0.001

Male (%)	60.5	77	0.024

Caucasian (%)	90.5	83.6	0.185

DM^2 ^(%)	15.2	22.9	0.211

Hypertension (%)	21.8	32.8	0.104

CVD^3 ^(%)	4.1	13.1	0.018

Cirrhosis (%)	11.1	9.8	0.955

HIV infection^4 ^(%)	12.3	13.1	0.957

Neoplasm (%)	5.3	13.1	0.063

APACHE II score^5^	16.9 (7.5)	24.2 (7.4)	< 0.0001

Nonrenal APACHE II score^6^	16.3 (7.2)	20.5 (7.5)	< 0.0001

Baseline SCr (mg/dL)^7^	0.8 (0.2)	1.1 (0.4)	< 0.0001

Septic shock (%)	18.1	55.7	< 0.0001

MV^8 ^(%)	32.5	47.5	0.041

Length of hospital stay (days)	10.7 (13)	12.8 (9.2)	0.233

^1^AKI-acute kidney injury. ^2^DM-diabetes mellitus. ^3^CVD-cardiovascular disease includes chronic heart failure and coronary artery disease. ^4^HIV infection-human immunodeficiency virus infection. ^5^APACHE II score-acute physiology and chronic health evaluation, version II score. ^6^Nonrenal APACHE II score-acute physiology and chronic health evaluation, version II score not including points for kidney insufficiency. ^7^Baseline SCr-baseline serum creatinine. ^8^MV-need for mechanical ventilation.

**Table 3:** Baseline characteristics of the hospital surviving septic patients with known or unknown follow-up.

Variable	Known follow-up (n = 234)	Unknown follow-up (n = 70)	***P***
Mean age (years)	51.2 (18.2)	54.2 (17.9)	0.234

Male (%)	63.7	64.3	0.961

Caucasian (%)	89.7	87.1	0.693

DM^1 ^(%)	15.8	20	0.522

Hypertension (%)	24.3	22.9	0.921

CVD^2 ^(%)	6	5.7	0.838

Cirrhosis (%)	10.7	11.4	0.258

HIV infection^3 ^(%)	13.7	8.6	0.354

Neoplasm (%)	1	0	0.371

APACHE II score^4^	18.3 (8.2)	18.7 (7.2)	0.751

Nonrenal APACHE II score^5^	17 (7.6)	17.5 (6.9)	0.644

Baseline SCr (mg/dL)^6^	0.8 (0.3)	0.8 (0.3)	0.922

Septic shock (%)	24.8	28.6	0.631

MV^7 ^(%)	35	37.1	0.857

AKI^8 ^(%)	20.5	18.6	0.853

Class R (%)	5.5	1.4	0.270

Class I (%)	6.8	6	0.410

Class F (%)	8.9	8.6	0.873

RRT^9 ^(%)	29.2	15.4	0.518

Complete RFR^10 ^(%)	87.5	100	0.414

Length of hospital stay (days)	10.9 (11.2)	11.8 (15.7)	0.589

^1^DM-diabetes mellitus. ^2^CVD-cardiovascular disease includes chronic heart failure and coronary artery disease. ^3^HIV infection-human immunodeficiency virus infection. ^4^APACHE II score-acute physiology and chronic health evaluation, version II score. ^5^Nonrenal APACHE II score-acute physiology and chronic health evaluation, version II score not including points for kidney insufficiency. ^6^Baseline SCr-baseline serum creatinine. ^7^MV-need for mechanical ventilation. ^8^AKI-acute kidney injury. ^9^RRT-need for renal replacement therapy. ^10^RFR-renal function recovery.

#### Definitions

Acute kidney injury was defined and categorized according to "Risk Injury Failure Loss of kidney function End-stage kidney disease" (RIFLE) classification, based on creatinine criteria [15]. Acute kidney injury was defined as an increase of baseline serum creatinine × 1.5, and in patients with baseline serum creatinine > 4 mg/dl it was also considered if there was an acute rise in serum creatinine of at least 0.5 mg/dl; Class R (Risk) was considered if there was an increase of baseline serum creatinine × 1.5; class I (Injury) was considered if there was an increase of baseline serum creatinine × 2; and Class F (Failure) was considered if there was an increase of baseline serum creatinine × 3, or in patients with baseline serum creatinine > 4 mg/dl if there was an acute rise in serum creatinine of at least 0.5 mg/dl. When pre-admission serum creatinine was available it was considered the baseline serum creatinine, and when it was unavailable baseline serum creatinine was estimated by the Modification of Diet in Renal Disease equation [16], as recommended (assuming a lower limit of the normal baseline glomerular filtration rate of 75 ml/min/1.73 m^2^) and previously applied [1,15]. In this unit, serum creatinine is determined at least once a day.

Sepsis was classified in accordance with the American College of Chest Physicians and the Society of Critical Care Medicine consensus [17] and Acute Physiology and Chronic Health Evaluation, version II (APACHE II) score was used to evaluate illness severity, and was calculated based on the worst variables recorded during the first 24 hours of ICU admission [18].

Complete renal function recovery was considered if the serum creatinine at hospital discharge with reference to baseline serum creatinine was lower than × 1.5, and in patients with baseline serum creatinine > 4 mg/dl complete renal function recovery was also considered if serum creatinine at hospital discharge was also lower than 0.5 mg/dl with reference to baseline serum creatinine.

#### Variables

Variables such as age, gender, race, comorbidity (diabetes mellitus, hypertension, cardiovascular disease which included chronic heart failure and coronary artery disease, cirrhosis, human immunodeficiency virus infection, and neoplasm), APACHE II score, need for mechanical ventilation, vasopressors use, daily serum creatinine as well as renal replacement therapy were collected from the unit database and patient medical charts.

#### Statistical analysis

Continuous variables are expressed as mean (standard deviation) and categorical variables as percentage of number of cases. Comparisons between patients with and without AKI were performed using the Student's t-test and the χ2-test, respectively, for continuous and categorical variables.

Only the first ICU presentation was analyzed for patients with multiple ICU admissions. Cumulative mortality curves were determined by the Kaplan-Meier method, and log-rank test was employed to analyze statistically significant differences between curves. Cox regression method was used to determine independent predictors of mortality. Risk factors were assessed with univariate analysis, and variables that were statistically significant (*P *< 0.05) in the univariate analysis were included in the multivariate analysis by applying a multiple forward stepwise Cox regression. The nonrenal APACHE II score was chosen as a covariate to control for multicollinearity between the AKI and scoring system that includes points for kidney insufficiency such as the APACHE II score. Data are presented as unadjusted and adjusted hazard ratios (HR) with 95% confidence intervals (CI). A two-tailed *P *value less than 0.05 was considered significant.

Analysis was performed with the statistical software package SPSS 16.0 for Windows.

## Results

### Acute kidney injury

Baseline serum creatinine was available in 293 patients (68.8%), and in the remaining cases (n = 133; 31.2%) it was estimated by the Modification of Diet in Renal Disease equation. Acute kidney injury occurred in 138 patients (32.4%), as follows: 26 patients (6.1%) were on class R, 34 patients (8%) were on class I, and 78 patients (18.3%) were on class F. Mean time to AKI development was 2.9 (4.7) days (1-19 days). As expected, patients with AKI were older, were more likely to have pre-existing cardiovascular disease, and had more severe disease as evaluated by total APACHE II score and nonrenal APACHE II score. Furthermore, baseline creatinine values were higher among patients with AKI, and septic shock and need for mechanical ventilation were more frequent in such patients (Table 1 and Table 2). Forty-one AKI patients (29.7%) did undergo renal replacement therapy (continuous venovenous hemodiafiltration in 23 patients, intermittent hemodialysis in 13 patients, and 5 patients were treated with both modalities). Patients with AKI had a greater risk for death within hospitalization (Table 1). Regarding hospital survivors lost to follow-up and with known follow-up it should be mentioned that they were similar in terms of age, gender, race, medical history, illness severity, baseline renal function, proportion of patients with AKI, proportion of AKI patients in the different RIFLE categories or undergoing renal replacement therapy, percentage of patients with septic shock or need for mechanical ventilation, and length of hospitalization (Table 3). The characteristics of the surviving and non surviving patients with AKI during ICU admission are described in Table 4.

**Table 4:** Baseline characteristics of hospital surviving and non surviving patients with acute kidney injury during Intensive Care Unit admission.

Variable	Non survivors (n = 77)	Survivors (n = 61)	***P***
Mean age (years)	58.8 (16.6)	58.8 (16.3)	0.991

Male (%)	68.8	77	0.378

Caucasian (%)	87	83.6	0.748

DM^1 ^(%)	11.7	22.9	0.125

Hypertension (%)	16.9	32.8	0.048

CVD^2 ^(%)	25.9	13.1	0.098

Cirrhosis (%)	10.4	9.8	0.885

HIV infection^3 ^(%)	20.8	13.1	0.340

Neoplasm (%)	10.4	13.1	0.819

APACHE II score^4^	30 (8.6)	24.2 (7.4)	< 0.0001

Nonrenal APACHE II score^5^	26.7 (8.5)	20.5 (7.5)	< 0.0001

Baseline SCr (mg/dL)^6^	1 (0.5)	1 (0.4)	0.379

Septic shock (%)	63.6	55.7	0.444

MV^7 ^(%)	68.8	47.5	0.019

RRT^8 ^(%)	32.5	26.2	0.543

^1^DM-diabetes mellitus. ^2^CVD-cardiovascular disease includes chronic heart failure and coronary artery disease. ^3^HIV infection-human immunodeficiency virus infection. ^4^APACHE II score-acute physiology and chronic health evaluation, version II score. ^5^Nonrenal APACHE II score-acute physiology and chronic health evaluation, version II score not including points for kidney insufficiency. ^6^Baseline SCr-baseline serum creatinine. ^7^MV-need for mechanical ventilation. ^8^RRT-need for renal replacement therapy.

#### Long-term outcome

**Figure 1:**
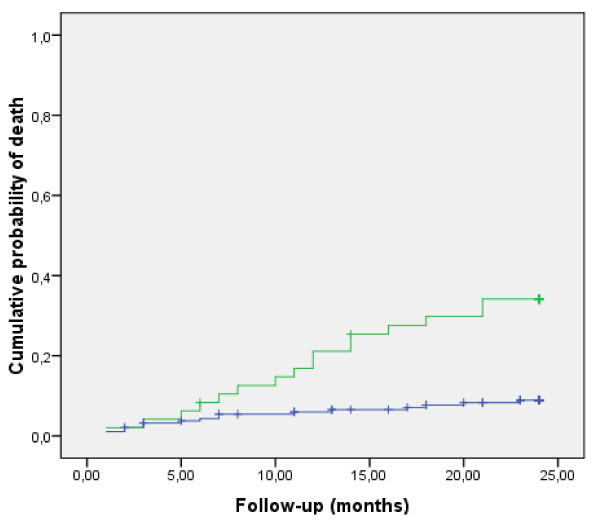
**Cumulative probability of death**. Cumulative probability of death in patients with (green line) and without (blue line) acute kidney injury (AKI) during previous Intensive Care Unit admission; log-rank test, *P *< 0.0001.

In this study, we focused on 234 patients [mean age: 51.2 (18.2) years, 149 male, 210 Caucasian, mean APACHE II score: 18.3 (8.2)] with sepsis, and analyzed their mortality at 2 years of follow-up as a cohort. Mean-follow-up was 21 (6.4) months. During this period, 32 patients (13.7%) died. The probability of death significantly differed among patients with AKI and without AKI during previous ICU admission (Figure 1). At 6 months, 1 and 2 years of follow-up, the cumulative probability of death of patients with AKI was 8.3, 16.9 and 34.2%, respectively, as compared with 2.2, 6 and 8.9% in patients without AKI (log-rank, *P *< 0.0001) (Figure 1). When we analyzed 2-year mortality in the different RIFLE categories, however, we did not find any statistically significant difference (class R, 7.7%; class I, 50%; class F, 38.1%; *P *= 0.055). In the univariate analysis, age (HR 1.4, 95% CI 1.2-1.7, *P *< 0.0001), as well as preexisting cardiovascular disease (HR 3.6, 95% CI 1.4-9.4, *P *= 0.009), illness severity as evaluated by nonrenal APACHE II (HR 1.3, 95% CI 1.1-1.6, *P *= 0.002), and AKI during previous ICU admission (HR 4.2, 95% CI 2.1-8.5, *P *< 0.0001) were associated with increased 2-year mortality, while gender, race, pre-existing hypertension, cirrhosis, HIV infection, neoplasm, and baseline glomerular filtration rate did not. In the multivariate analysis, however, only AKI during previous ICU admission (AHR 3.2, 95% CI 1.6-6.5, P = 0.001) and age (AHR 1.4, 95% CI 1.2-1.6, P < 0.0001) emerged as independent predictors of 2-year mortality (Table 5).

**Table 5:** Univariate and multivariate Cox regression analysis to determine predictors of 2-year mortality.

Variable	Unadjusted HR^1 ^(95% CI^3^)	***P***	AHR^2 ^(95% CI^3^)	***P***
Age (per 10 years)	1.4 (1.2-1.7)	< 0.0001	1.4 (1.2-1.6)	< 0.0001

Gender (male)	1.1 (0.5-2.3)	0.752		

Race (Caucasian)	3.8 (0.5-27.6)	0.192		

Diabetes mellitus	2.1 (0.9-4.6)	0.06		

Hypertension	1.7 (0.8-3.4)	0.177		

CVD^4^	3.6 (1.4-9.4)	0.009		

Cirrhosis	0.9 (0.3-2.8)	0.813		

HIV infection^5^	1.9 (0.9-4.6)	0.107		

Neoplasm (%)	2.5 (0.9-7.1)	0.088		

Baseline GFR^6 ^(per 10 ml/min/1.73 m2)	1.1 (0.9-1.3)	0.063		

Nonrenal APACHE II^7^(per 5 points)	1.3 (1.1-1.6)	0.002		

AKI^8^	4.2 (2.1-8.5)	< 0.0001	3.2 (1.6-6.5)	0.001

^1^HR-hazard ratios. ^2^AHR-adjusted hazard ratios. ^3^CI-confidence intervals. ^4^CVD-Cardiovascular disease includes chronic heart failure and coronary artery disease. ^5^HIV infection-human immunodeficiency virus infection. ^6^GFR-baseline glomerular filtration rate. ^7^Nonrenal APACHE II score-acute physiology and chronic health evaluation, version II score not including points for kidney insufficiency. ^8^AKI-acute kidney injury during previous ICU admission.

Of interest, 2-year mortality did not significantly differ between AKI patients who completely recovered renal function (n = 14, 33.3%) and those who did not (n = 2, 33.3%).

## Discussion

It has been shown that patients who survive AKI have a greater rate of long-term mortality and other adverse outcomes than patients who survive hospitalization without AKI, in varied settings, such as community/hospitalized patients, hospitalized patients, ICU patients, cardiac surgery, patients undergoing aortic stent placement or percutaneous coronary intervention, liver transplant and lung transplant [19].

Sepsis is a well-known risk factor for the development of AKI and nearly half of all patients with AKI have sepsis [8,9]. Furthermore, AKI is associated with short-term mortality among septic patients [8-14]. Despite the detrimental effect of AKI on short-term outcome, to our knowledge, the influence of AKI on long-term mortality of septic patients has not yet been assessed.

We conducted a retrospective study of a cohort of 234 hospital surviving septic patients, in order to determine the impact of AKI during ICU admission on 2-year mortality. We found that AKI had a negative impact on 2-year mortality of patients with sepsis. As compared with patients without acute renal impairment, patients with AKI had an increased probability of death of 25.3%, which corresponded to a 3.2-fold risk of death. These findings expand on results from previous studies that showed the increased risk of short-term death associated with AKI in septic patients [8-14].

The mechanism by which AKI contributes to decreased long-term survival is not completely understood. After an episode of AKI, it is likely that there is failure to resolve renal structure and function adequately [20-22]. Acute kidney injury itself may increase the risk of subsequent events and decrease kidney reserve leading to an increased risk of chronic kidney disease. In fact, despite we do not have serial values of blood pressure, serum creatinine and proteinuria, it has been shown that chronic kidney disease with subsequent hypertension, proteinuria and increased cardiovascular disease has been appointed as a possible cause of poor long-term outcome among AKI patients [23]. Moreover, volume overload, coagulation abnormalities, an increased incidence of sepsis with multi-organ failure, and cytokine or immune-mediated major organ dysfunction are other possible explanations for poor survival among AKI patients [24,25].

In the present study, mortality did not differ among AKI patients with and without complete renal function recovery. These results should be interpreted with extreme caution, since the number of patients may be insufficient to detect a difference on outcome. However, the similar outcome between AKI patients who did or not recover renal function could also be explained by the permanent injury to other vital organs caused by AKI, thus affecting future survival, despite the reversible nature of clinical AKI in which serum creatinine level returns to baseline after the episode [24-26]. Therefore, our results could also suggest that despite patients with septic AKI are prone to recover renal function [9,14], they should be closely monitored after discharge.

Our study has several limitations. First, the single-centre nature of the study largely limits its generalizability, and its retrospective design with a relatively small cohort of patients did not allow the evaluation of other confounders with prognostic importance. Second, baseline serum creatinine was available only in 68.8% of patients, while in the remaining cases (31.2%) it was estimated by the Modification of Diet in Renal Disease equation, as it has been recommended and previously applied [1,15]. Regarding this, it should be stressed that the use of Modification of Diet in Renal Disease equation to estimate baseline serum creatinine, despite performing reasonable well when basal glomerular filtration rate is normal or near normal, could overestimate the incidence of AKI in chronic kidney disease patients [27]. Third, the evolution for chronic kidney disease and end-stage renal disease after AKI, as well as the causes of death were unattainable. Fourth, outcome data were available only in 77% of patients. Regarding this issue, it is important to mention that hospital survivors lost to follow-up and with known follow-up were similar in terms of age, gender, race, medical history, illness severity, baseline renal function, proportion of patients with AKI, proportion of AKI patients in the different RIFLE categories or undergoing renal replacement therapy, percentage of patients with septic shock or need for mechanical ventilation, and length of hospitalization. Taking into consideration the similar characteristics of hospital survivors lost to follow-up and with known follow-up, we think that it is not expectable that substantially different results would be achieved if the outcome data of all surviving patients was available. In fact, our results are in line with those focusing on the effect of AKI on long-term outcome in other settings [19], and likely, AKI can also exert a negative influence on long-term outcome of patients with sepsis. Finally, the number of patients in the different AKI categories was too small to determine the role of severity of AKI on long-term outcome.

Despite the aforementioned constraints, our study has the main virtue of being, to our knowledge, the first study reporting the negative impact of AKI on long-term mortality of patients with sepsis, a specific setting where AKI is pathophysiological and clinically distinct from AKI of other etiologies [9,28]. The appropriate management of the patients with sepsis through early goal-directed resuscitation, early administration of broad-spectrum antibiotic therapy, therapy with vasopressor agents (when fluid challenge fails to restore adequate blood pressure and organ perfusion), use of recombinant activated protein C (in patients with severe sepsis and high risk for death), use of low-tidal volume ventilation and blood glucose control is essential to prevent or limit AKI severity and, as such, could improve the long-term outcome of septic patients after hospital discharge [29].

## Conclusions

Our results suggest that AKI negatively influences long-term mortality of patients with sepsis. Despite septic patients who develop AKI and survive their acute episode have greater likelihood for renal function recovery the negative impact of AKI on long-term mortality deserves an additional effort to closely monitor them after hospital discharge.

## Abbreviations

AHR: adjusted hazard ratios; AKI: acute kidney injury; ANOVA: one-way analysis of variance; APACHE II score: acute physiology and chronic health evaluation, version II score; CI: confidence intervals; CVD: cardiovascular disease; DM: diabetes mellitus; GFR: glomerular filtration rate; HIV: human immunodeficiency virus; HR: hazard ratios; ICU: intensive care unit; MV: need for mechanical ventilation; RIFLE: Risk Injury Failure Loss of kidney function End-stage kidney disease; RRT: need for renal replacement therapy; SCr: serum creatinine.

## Competing interests

The authors declare that they have no competing interests.

## Authors' contributions

JAL participated in the conception of the study, analysis and interpretation of data, and in drafting the manuscript. PF, SJ, CR and CS participated in the collection of data. AP and JN participated in the conception of the study, and in revising the manuscript. FA and AGC provided intellectual content of critical importance to the work described. All authors gave final approval of the version to be published.

## Pre-publication history

The pre-publication history for this paper can be accessed here:

http://www.biomedcentral.com/1471-2369/11/9/prepub
